# Identification of hub genes and biological mechanisms underlying the pathogenesis of asthenozoospermia and chronic epididymitis

**DOI:** 10.3389/fgene.2023.1110218

**Published:** 2023-04-21

**Authors:** Yinwei Chen, Taotao Sun, Longjie Gu, Song Ouyang, Kang Liu, Penghui Yuan, Chang Liu

**Affiliations:** ^1^ Reproductive Medicine Center, Tongji Hospital, Tongji Medical College, Huazhong University of Science and Technology, Wuhan, Hubei, China; ^2^ Department of Urology, Tongji Hospital, Tongji Medical College, Huazhong University of Science and Technology, Wuhan, Hubei, China; ^3^ Department of Urology, First Affiliated Hospital, School of Medicine, Shihezi University, Shihezi, Xinjiang, China; ^4^ Department of Urology, The First Affiliated Hospital of Zhengzhou University, Zhengzhou, Henan, China; ^5^ Reproductive Medicine Center, Nanjing Drum Tower Hospital, The Affiliated Hospital of Nanjing University Medical School, Nanjing, Jiangsu, China

**Keywords:** asthenozoospermia, chronic epididymitis, immune infiltration, miRNA, bioinformatics

## Abstract

**Objective:** Asthenozoospermia (AZS) is one of the most common causes of male fertility, affecting family wellbeing and population growth. Chronic epididymitis (CE) is a common and lingering inflammatory disease in the scrotum. Inflammation in the epididymis has a severe impact on sperm motility. This study aimed to explore the genetic profile and critical pathways involved in the pathological mechanisms of AZS and CE, and discover potential biomarkers.

**Methods:** Genomic datasets of AZS and CE were obtained from the Gene Expression Omnibus (GEO) database, and relevant differentially expressed genes (DEGs) were identified. GO and pathway enrichment analyses, construction of a protein-protein interaction network, and receiver operator characteristic curve analysis were conducted. The expression profile of hub genes was validated in immunohistochemical data and testicular cell data. Immune infiltration, miRNA-hub gene interactions, and gene-disease interactions were explored. The mRNA levels of hub genes were further measured by qRT-PCR.

**Results:** A total of 109 DEGs were identified between the AZS/CE and healthy control groups. Pathways of the immune system, neutrophil degranulation, and interleukin-4 and interleukin-13 signaling were enriched in AZS and CE. Five hub genes (*CD300LB, CMKLR1, CCR4, B3GALT5,* and *CTSK*) were selected, and their diagnostic values were validated in AZS, CE, and independent validation sets (area under the curve >0.7). Furthermore, the five-hub gene signature was well characterized in testicular immunohistochemical staining and testicular cells from healthy controls. Immune infiltration analysis showed that infiltration of CD8^+^ cells and T helper cells was significantly related to the expression level of five hub genes. In addition, a miRNA-hub gene network and interaction of other diseases were displayed. The mRNA levels of hub genes (*CD300LB, CMKLR1, CCR4,* and *B3GALT5*) were significantly elevated in the patient group. The mRNA level of CTSK also showed a similar trend.

**Conclusion:** Our study uncovered the genetic profile involved in AZS and CE, and elucidated enriched pathways and molecular associations between hub genes and immune infiltration. This finding provides novel insight into the common pathogenesis of both diseases as well as the potential biomarkers for CE-associated AZS.

## 1 Introduction

Asthenozoospermia (AZS) refers to sperm motility of less than 42% or less than 30% (fifth percentile in the reference population) with progressive motility according to the WHO manual for human semen (World Health Organization, 2021). It is one of the most common causes of infertility in men, and a majority of instances (up to 80%) of male infertility are caused by AZS which differs in severity (Tu et al., 2020). In most conditions, asthenozoospermia is often accompanied by oligospermia and teratozoospermia. Therefore, asthenozoospermia often acts as one of the important clinical indications suitable for assisted reproductive technology, which causes a heavy burden on family wellbeing and the social healthcare system. Currently, the etiology of AZS is not yet fully understood. Accumulating evidence has shown that many factors can lead to poor sperm motility, including infection/inflammation, varicoceles, antisperm antibodies, damaged sperm structures, and genomic abnormalities (Dimitrov et al., 1994; Bonanno et al., 2016; Lehti and Sironen, 2017). Clarifying the mechanism of AZS is the key to effectively restoring sperm motility.

Infection/inflammation of the male genital tract is one of the main causes of AZS. Studies have found that an increased number of leukocytes in semen releases large amounts of reactive oxygen species (ROS), further resulting in low sperm motility and DNA integrity (Haidl et al., 2015; Li et al., 2020; Liu et al., 2021). The protective effects of immune modulators (e.g., beta-glucan) and antioxidants against inflammatory processes could improve sperm maturation and migration for patients with AZS and leukocytosis, further helping them with fertility-related issues (Piomboni et al., 2008). Treatment for AZS ameliorating inflammation in the reproductive tractshould be further explored.

Chronic epididymitis (CE) is a common inflammatory disease in the scrotum that has clinical manifestations including local pain, swelling, lumps, nodules, and male infertility (McConaghy and Panchal, 2016). The National Academy of Medicine found that 600,000 patients are diagnosed with epididymitis in nearly 1.5 billion visits each year (Nickel et al., 2005). Although antibiotics and anti-inflammatory drugs serve as common therapeutic drugs for CE, the recurrent and lingering characteristics of this disease make treatment challenging. Studies have shown that male infertility affects up to 40% of patients with epididymitis (Michel et al., 2015; Zhao H. et al., 2020), and a high level of seminal plasma leptin is regarded as one of the main contributors to the reduction in sperm viability and migration in patients with CE (Lin et al., 2020). However, there are still many unknowns regarding the mechanism of AZS due to the influence of CE.

With the advancement of sequencing techniques, bioinformatic analysis has played a major role in uncovering the molecular mechanisms and genetic alterations underlying the development of various diseases (Campbell et al., 2008; Wang et al., 2009). Traditional gene-by-gene approaches in research are no longer sufficient to meet the requirement of massive amounts of genetic data. In particular, bioinformatic tools have shown advantages in cross-sectional studies between diseases, not only helping researchers to find critical regulators more accurately, but also saving considerable time and economic costs. For example, Luo et al. identified twelve significant hub genes (*CMPK2, TYMS, etc.*) as potential diagnostic markers for COVID-19 and primary Sjogren’s syndrome by DEG analysis (Luo and Zhou, 2022). By immunological bioinformatic analysis, Yao et al. reported that neutrophil migration with high expression of S100A8, S100A9, S100A12 and CXCR2 plays a central role in the development of Crohn’s disease and peripheral arterial disease (Yao et al., 2022). Nevertheless, the above effective bioinformatic methods have not been used to study the pathogenesis of AZS and CE.

In the present study, we systematically analyzed the hub genes and biological processes involved in asthenozoospermia and chronic epididymitis via multiomics evaluation. The hub gene signature with a specific expression pattern was validated in the experimental data, and its immune infiltration correlation was depicted in detail. Our study will aid in revealing the common mechanisms and critical regulators in both AZS and CE.

## 2 Methods and materials

### 2.1 AZS and CE dataset collection

We searched “asthenozoospermia” or “chronic epididymitis” in the GEO database (https://www.ncbi.nlm.nih.gov/geo/). After careful screening, the AZS datasets (GSE160749 and GSE22331) and CE dataset (GSE199903) were downloaded from the GEO database. GSE160769 contained genetic information on five AZS patients and six healthy controls. The GSE22331 dataset contained genetic information from 30 AZS patients and 30 healthy controls. Transcriptome data from an independent validation set including 3 AZS patients and 9 healthy controls were obtained from GSE26881. All AZS samples did not exclude other relevant description. Additionally, the single-cell transcriptome of the testis was obtained from GSE149512. Detailed information on the above GEO datasets is shown in Supplementary Table S1.

### 2.2 DEG analysis

All expression matrices were regularly normalized and log2 transformed (if needed) by using the R package “limma” (Ritchie et al., 2015). For obtain the integrated AZS dataset, two GEO datasets (GSE160749 and GSE22331) were continually merged using Strawberry-Perl software (Version 5.30). The batch effect between arrays was then eliminated using the R package “sva” (Leek et al., 2012). The differentially expressed genes (DEGs) between the AZS and control groups were calculated based on |log2-fold change| (|log2FC|) > 0.5 and *p*-value <0.05. The same analytical approach was applied to DEGs between the CE and control groups. Then, the intersection of AZS DEGs and CE DEGs was performed to obtain intersecting DEGs involved in the two diseases.

### 2.3 Functional enrichment analysis

To further depict the biological function and pathways implicated in the intersected genes, we performed Gene Ontology (GO) analysis and REACTOME pathway analysis by using the DAVID Bioinformatics Microarray online tool (https://david.ncifcrf.gov/) (Huang et al., 2007). GO analysis includes biological process (BP), cellular component (CC), and molecular function (MF). Visualization of GO clusters and pathway clusters was performed by R package “ggplot2” (Walter et al., 2015). *p*-value <0.05 was set as the cutoff criterion.

### 2.4 PPI network analysis

The protein interaction network was analyzed via the Search Tool for the Retrieval of Interacting Genes (STRING) database (http://string-db.org). For more potential interactions, a confidence score >0.15 was set as the cutoff criterion. Visualization of the PPI network was carried out using Cytoscape software (Version 3.7). Moreover, the MCODE plugin was used to obtain the top three gene clusters, and the cytoHubba plugin was used to obtain the top 20 genes with the highest MCC score.

### 2.5 Diagnostic analysis in different datasets

We first narrowed down the number of intersected genes, and defined the co-DEGs as the intersected genes with the same expression trend (upregulation and downregulation) in the AZS and CE datasets. Subsequently, to assess the efficacy of these co-DEGs in predicting AZS and CE, we created receiver operator characteristic (ROC) curves using the R package “timeROC” (https://cran.r-project.org/web/packages/timeROC/) (Blanche et al., 2013) and evaluated the area under the curve (AUC) of these co-DEGs. The co-DEGs with AUCs >0.7 in both the AZS dataset and CE dataset were selected. Among them, hub genes were further selected and validated in the independent validation set (GSE26881) based on AUC >0.7. Visualization was carried out by the R package “ggplot2”.

### 2.6 Immunohistochemical analysis of hub genes in the testis

The expression localization of hub genes was evaluated in immunohistochemical data downloaded from the Human Protein Atlas database (HPA) (https://www.proteinatlas.org/). Resource addresses of immunohistochemical data are shown in Supplementary Table S2.

### 2.7 Single-cell expression analysis of hub genes

To identify the role of hub genes in testicular function, we used GSE149512 which contained the single-cell transcriptome of testes from ten males ranging from infants to adults, and GSE211115 which contained the single-cell transcriptome of testes from nine male mice at different age stages. The expression pattern of hub genes was analyzed and visualized in the Male Health Atlas database (http://www.malehealthatlas.cn/) (Zhao L. et al., 2020).

### 2.8 Immune infiltration analysis

Single-sample gene set enrichment analysis GSEA (ssGSEA), an extension of GSEA, computes enrichment scores separately for each sample-gene set pairing. This method is widely used in the calculation of immune infiltration in genetic expression datasets (Finotello and Trajanoski, 2018). We performed immune infiltration analysis for the AZS dataset and CE dataset, and calculated the enrichment score of each immune cell and function for each sample. Correlations among the above immune proportions and correlations between the hub genes and immune proportions were carried out using Spearman’s method. A *p*-value <0.05 was regarded as statistically significant.

### 2.9 Hub gene interaction

For identification of the interacting genes of hub genes, a gene-gene interaction network was constructed via the online tool GeneMANIA (http://genemania.org/). The network showed the association in the fields of physical interaction, coexpression, predicted, colocalization, genetic interactions, pathway, and shared protein domains.

### 2.10 MiRNA-hub gene network prediction

According to the five hub genes, the online tool miRcode (http://www.mircode.org/) (Jeggari et al., 2012) was used to predict target miRNAs in humans with default criteria. Visualization of the hub gene-miRNA network was carried out by Cytoscape software. With the use of the cytoHubba plugin, the top ten miRNAs with the highest MCC score were obtained.

### 2.11 Disease association analysis

To identify the association between hub genes and disease, we used the Comparative Toxicogenomics Database (CTD, http://ctdbase.org/) (Davis et al., 2017) to generate their relevancy. Male reproductive system diseases were screened, and the Inference Score was limited to >10.

### 2.12 Subjects and clinical examinations

Ten subjects attending Tongji Hospital were recruited in the present study. Among them, five patients were diagnosed with AZS and CE, and five subjects were healthy controls. According to the WHO laboratory manual (World Health Organization, 2021), sperm motility of less than 42% or less than 30% with progressive motility in at least two semen exams was required for AZS. Patients were diagnosed with chronic epididymitis based on the criteria requirements of CE (McConaghy and Panchal, 2016). The Institutional Research Ethics Committee of Huazhong University of Science and Technology approved our study (REC No. (2017(04)). We obtained written consent from each subject, and all procedures adhered to the Helsinki Declaration.

The medical histories of patients with AZS and CE and controls were collected. We excluded subjects with a history of varicocele, diabetes mellitus, scrotal trauma, and exposure to radiation and environmental pollutants. Chemiluminescence immunoassays (Beckman Coulter, Fullerton, United States) were used to measure the levels of FSH, LH, total T, and estradiol (E_2_) in the serum. Seminal elastase was detected using enzyme-linked immunosorbent assays (ELISAs) (Milenia, Bad Nauheim, Germany). We performed an ultrasound examination for male genital condition (850 system, Hitachi, Beijing, China). Semen was harvested by masturbation after 3–5 days of abstinence, and sperm processing and testing were in strict compliance with the WHO laboratory manual (World Health Organization, 2021). We used a Percoll gradient (Solarbio, Beijing, China) and centrifugation for 20 min at 1,000 g to eliminate seminal plasma and purify sperm samples. We washed them with PBS twice, and kept sperm pellets for later measurement.

### 2.13 mRNA measurement by qRT-PCR

We used RNA-easyTM Isolation Reagent (Vazyme, Nanjing, China) to extract RNA from sperm samples according to the manufacturer’s instructions. And cDNA was synthesized with the Hifair^®^ Ⅱ 1st Strand cDNA Synthesis Kit (Yeasen, Shanghai, China). The detailed procedures of quantitative real-time polymerase chain reaction (qRT-PCR) were described previously (Chen et al., 2021). Then, primers were synthesized (Tsingke, Wuhan, China), and sequence information is shown in Supplementary Table S3. The 2^−ΔΔct^ relative quantification algorithm was utilized to evaluate the mRNA levels of hub genes in each sample.

### 2.14 Statistical analysis

The difference in mRNA levels was compared using Student’s *t*-test. All statistical analyses we used in the present study were carried out by R software (version 4.1.3, https://www.r-project.org/) and SPSS version 23.0 (IBM Corporation, Armonk, United States). Statistical significance was defined as a *p*-value less than 0.05.

## 3 Results

### 3.1 Identification of DEGs in AZS and CE

**FIGURE 1:**
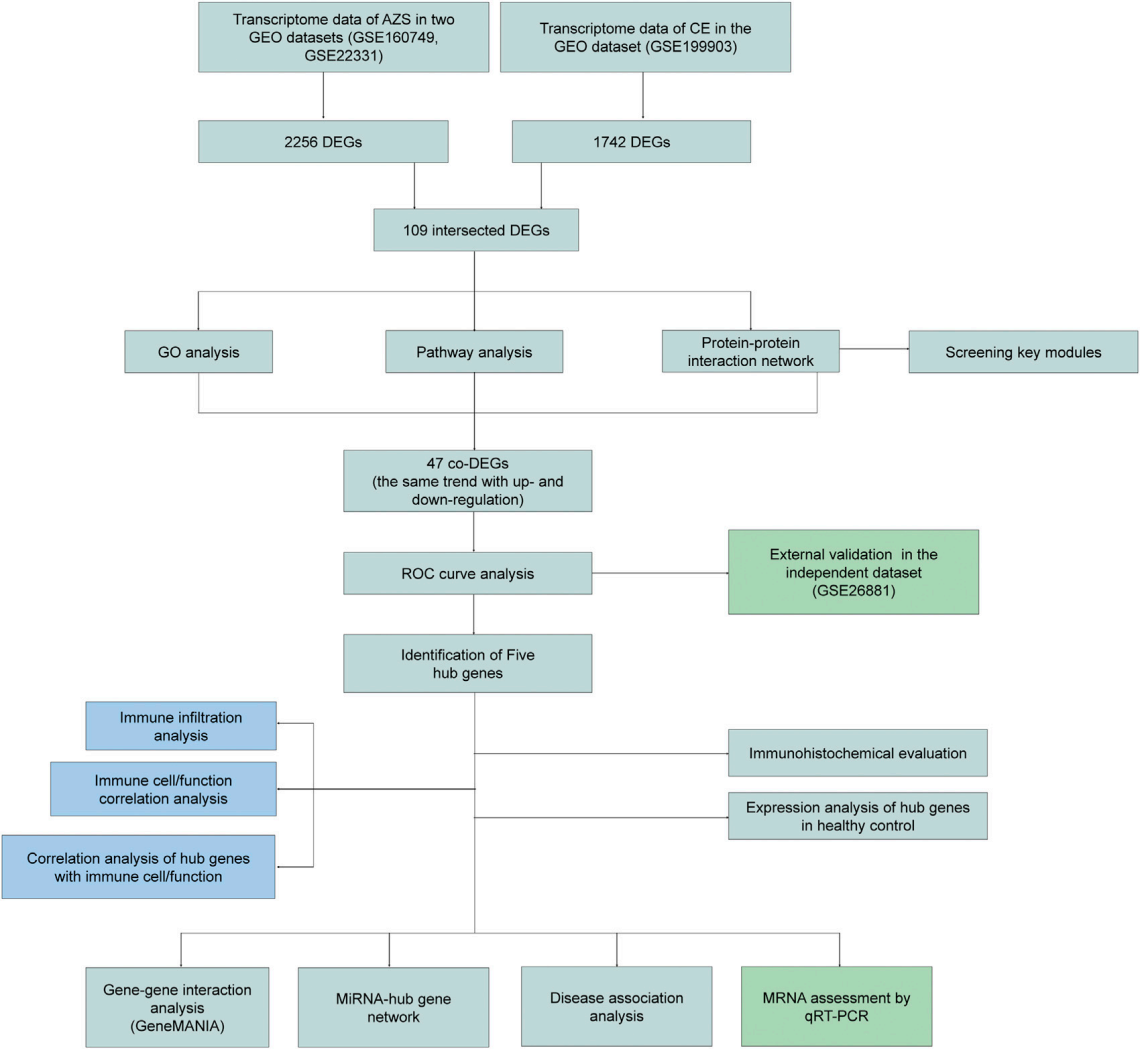
The flow diagram of study design. AZS: asthenozoospermia; CE: chronic epididymitis; GEO: Gene Expression Omnibus; GSE: GEO Series; DEGs: Differentially expressed genes; co-DEGs: co-expressed differentially expressed genes; GO: Gene Ontology; ROC: Receiver Operating Characteristic; qRT-PCR: quantitative real-time polymerase chain reaction.

**FIGURE 2:**
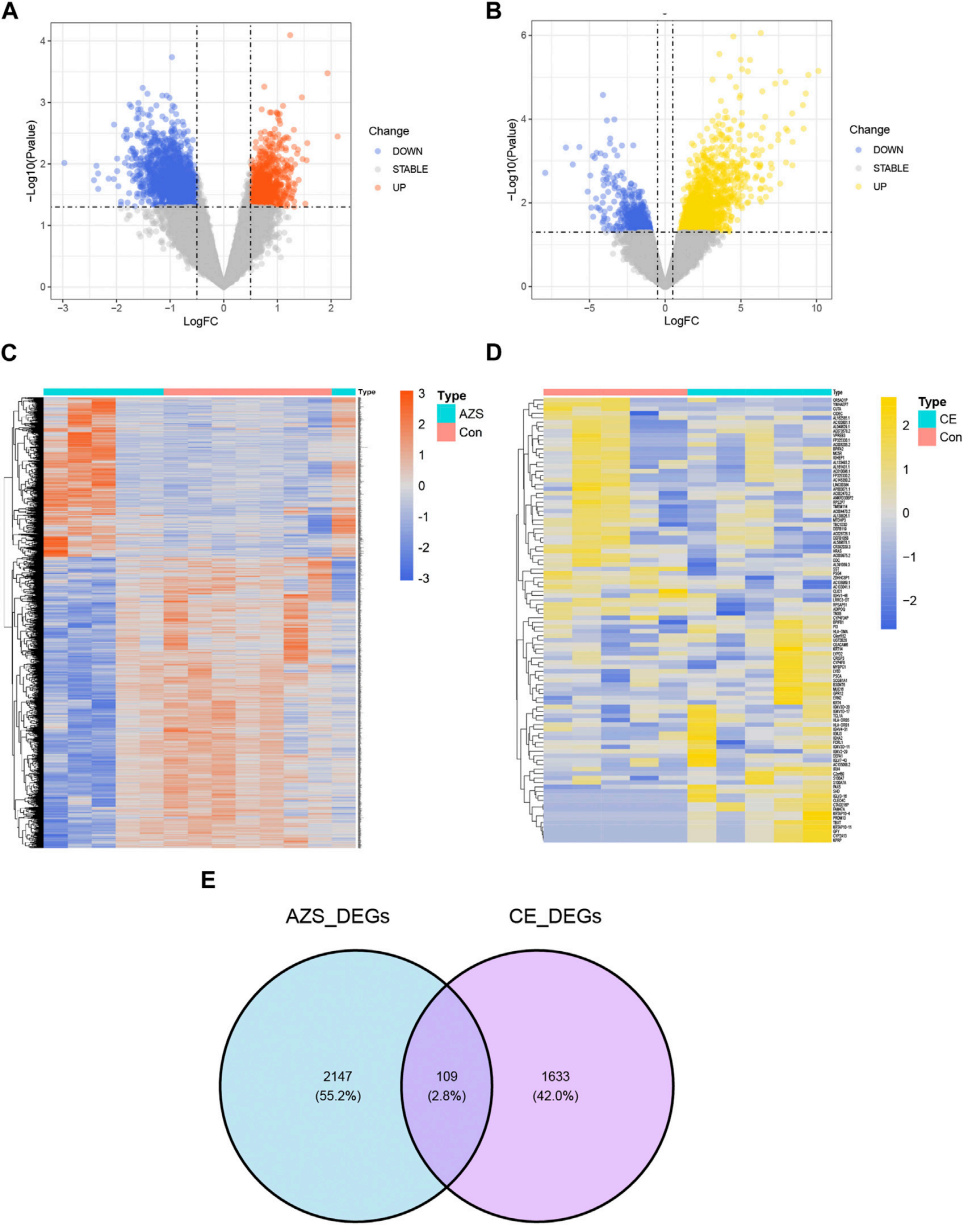
Differently expressed genes in the integrated AZS GEO dataset (GSE160749 and GSE22331) and the CE GEO dataset (GSE199903). **(A)** In the volcano map of DEGs in the integrated AZS GEO dataset, blue nodes denote downregulation, orange nodes denote upregulation, and grey nodes denote no significant difference. **(B)** In the volcano map of DEGs in the CE GEO dataset, blue nodes denote downregulation, yellow nodes denote upregulation, and grey nodes denote no significant difference. **(C)** The heatmap of DEGs in AZS. **(D)** The heatmap of DEGs in CE. **(E)** Venn diagram from intersected genes of the AZS DEGs and CE DEGs. AZS: asthenozoospermia; CE: chronic epididymitis; Con: Control; FC: fold change; DEGs: Differentially expressed genes.

The research design of this study is shown in Figure 1. With the criteria of *p*-value <0.05 and |log2FC| > 0.5, a total of 2,256 DEGs were selected from the AZS dataset: 800 upregulated DEGs and 1,456 downregulated DEGs. In the CE dataset, a total of 1742 DEGs were selected: 1,271 upregulated DEGs and 471 downregulated DEGs. The expression patterns of both DEG sets are shown in volcano plots and heatmaps (Figures 2A–D). To obtain the DEGs that may participate in the development of these two diseases, we took the intersection of AZS DEGs and CE DEGs. A Venn diagram showed that 109 DEGs were expressed simultaneously in the two DEG groups (Figure 2E).

### 3.2 Functional enrichment of intersected DEGs

**FIGURE 3:**
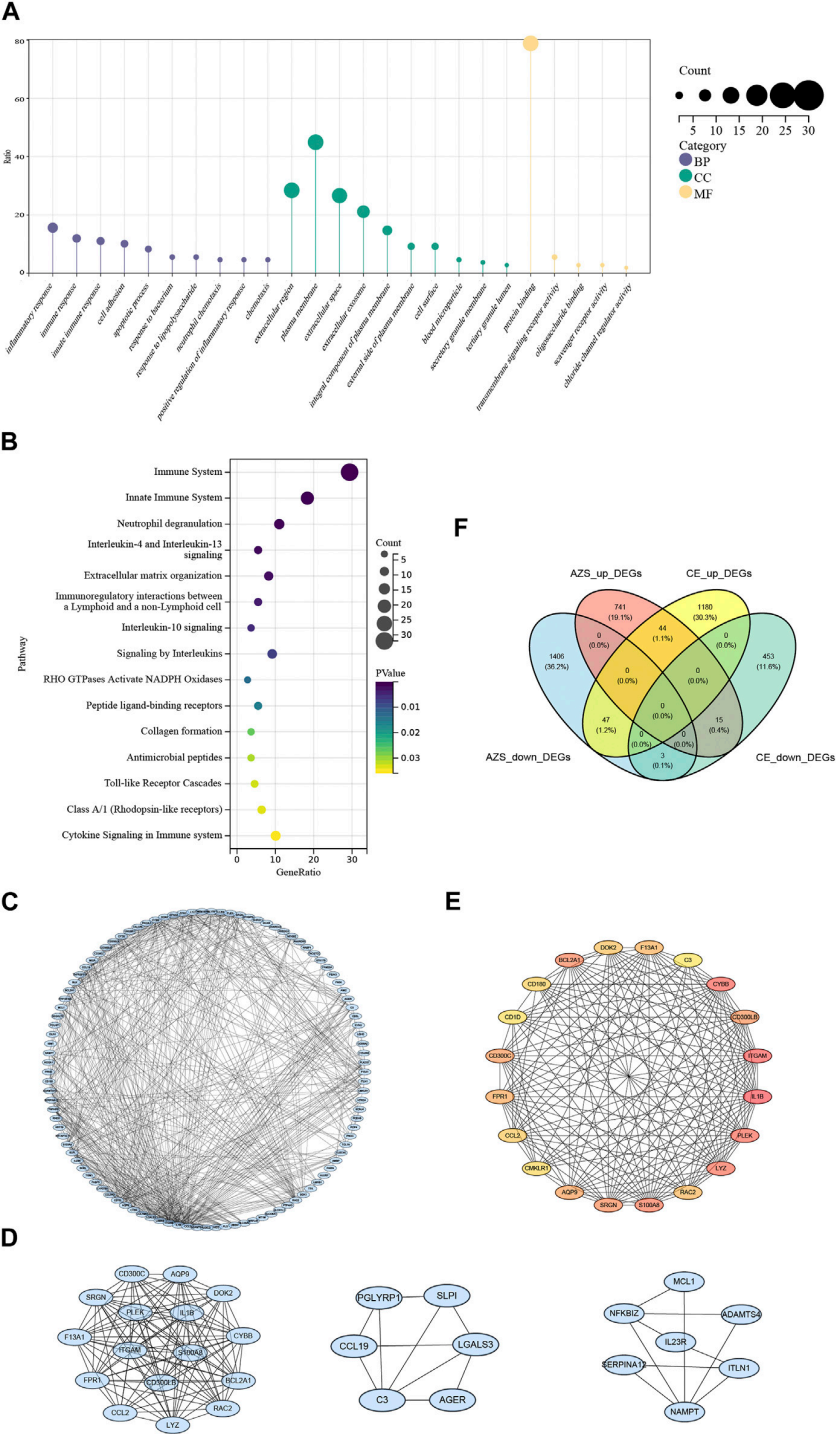
Functional enrichment results and protein-protein interaction network of intersected DEGs. **(A)** GO cluster enrichment in BP, CC, and MF. **(B)** Pathway enrichment for intersected DEGs. **(C)** The protein-protein interaction (PPI) network for intersected DEGs. **(D)** Top three key modules screened from PPI network. **(E)** The top 20 DEGs interaction network screened from the PPI network based on MCODE. **(F)** Venn diagram from co-DEGs of the AZS DEGs and CE DEGs. Co-DEGs denote the intersected DEGs with the same expression trend (upregulation and downregulation) in the AZS dataset and CE dataset. AZS: asthenozoospermia; CE: chronic epididymitis; DEGs: differently expressed genes; co-DEGs: co-expressed differentially expressed genes; GO: Gene Ontology; BP: biological process; CC: cellular component; MF: molecular function; PPI: protein-protein interaction.

To investigate inherent biological information in more detail, we performed GO and pathway analysis for the intersected DEGs. As shown in Figure 3A, for biological process, GO terms were enriched in inflammatory response, immune response, and cell adhesion. For cellular components, GO terms were enriched in extracellular region, plasma membrane, and extracellular space. For molecular function, GO terms were enriched in protein binding, transmembrane signaling receptor activity, and oligosaccharide binding. In addition, REACTOME pathway analysis showed that the DEGs were mostly enriched in the immune system, neutrophil degranulation, and interleukin-4 and interleukin-13 signaling (Figure 3B). The functional enrichment results indicated that the 109 intersecting DEGs had a close relationship with immune-related biological mechanisms.

### 3.3 PPI network construction

To further explain the relationships between the intersecting genes, we used the STRING database to identify protein interactions. Figure 3C displays the PPI network, which consisted of 587 interactions. Moreover, the top three DEG clusters were calculated based on the MCODE algorithm (Figure 3D). According to the MCC algorithm, the top 20 DEGs were obtained to show their interaction in Figure 3E. Herein, *CMKLR1, CD300LB, IL1B,* and *C3* (complement C3) were found in this top 20 DEGs interaction, and these genes are strongly associated with the activation of immune responses and the release of inflammatory mediators.

### 3.4 Diagnostic value of hub genes and further validation

**FIGURE 4:**
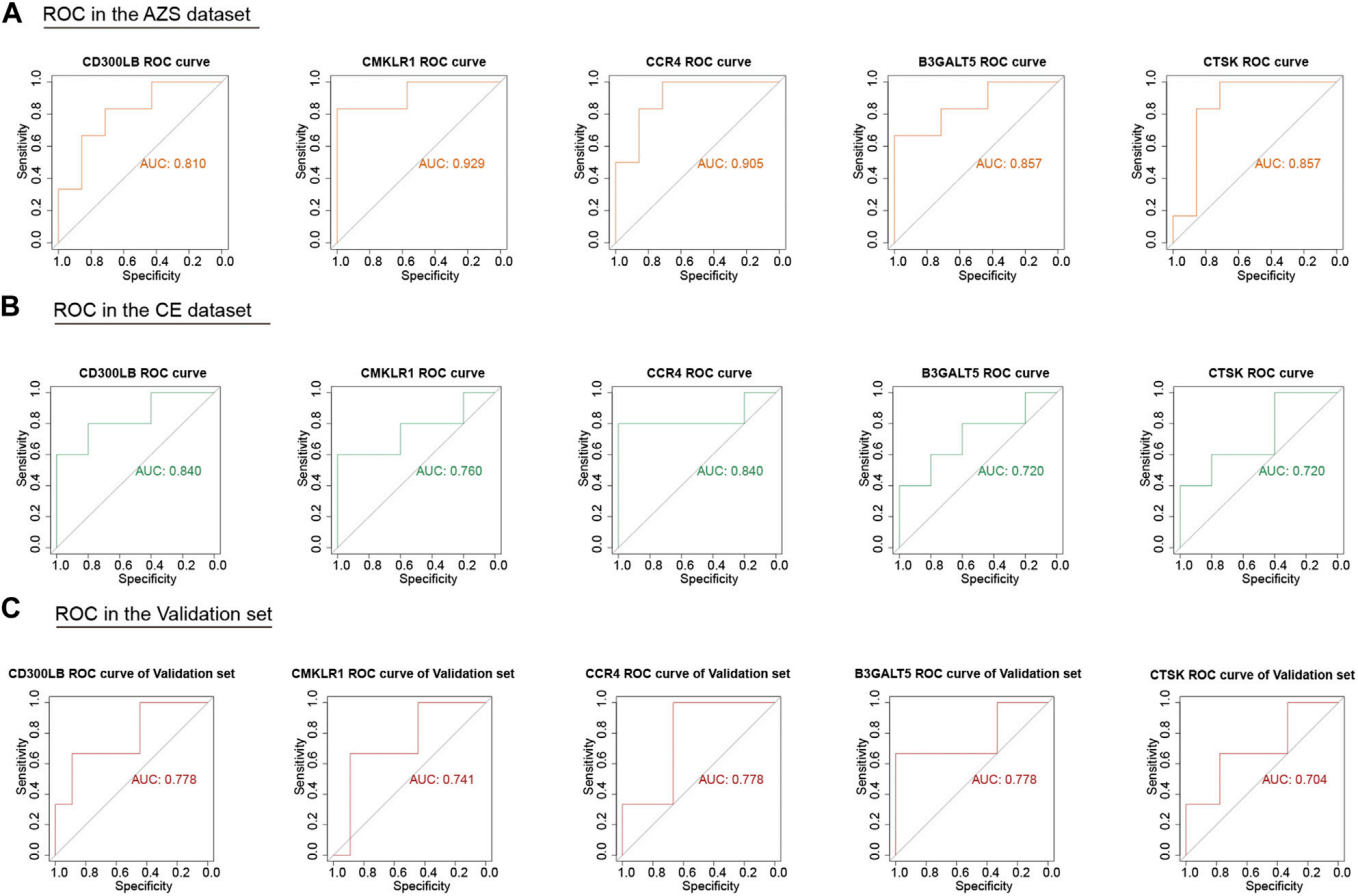
ROC analysis of five hub genes (*CD300LB, CMKLR1, CCR4, B3GALT5,* and *CTSK*) in the AZS, CE, and validation dataset. **(A)** ROC analysis of five hub genes in the AZS dataset. **(B)** ROC analysis of five hub genes in the CE dataset. **(C)** ROC analysis of five hub genes in the validation set (GSE26881). AZS: asthenozoospermia; CE: chronic epididymitis; ROC: Receiver Operating Characteristic; AUC: Area Under the Curve.

Although we screened out a batch of DEGs, the number in this batch was too large. To narrow down the range of the DEGs, we further selected 47 coexpressed DEGs (co-DEGs) with the same expression trend (upregulation and downregulation) in the AZS dataset and CE dataset (Figure 3F). With the use of the R package “pROC,” we explored the above genes with high accuracy of the diagnostic characteristics. The ROC results of the AZS dataset showed that five genes (*CD300LB, CMKLR1, CCR4, B3GALT5,* and *CTSK*) had good diagnostic values (AUC >0.7) to discriminate the samples from the patients with AZS and healthy controls. Similarly, the diagnostic values of the five genes were also good in the CE dataset (AUC >0.7) (Figures 4A,B). Based on their good diagnostic performance, these five genes were regarded as hub genes in the present study.

### 3.5 External data validation of hub genes

To further evaluate the important role of the five hub genes, we downloaded another AZS dataset as the independent validation set. After ROC evaluation, Figure 4C shows that the AUC values of the five hub genes were all greater than 0.7 in the validation set. These results indicated that the five hub genes have the potential to be biomarkers for AZS and CE.

### 3.6 Expression of hub genes in the testis (the HPA database)

**FIGURE 5:**
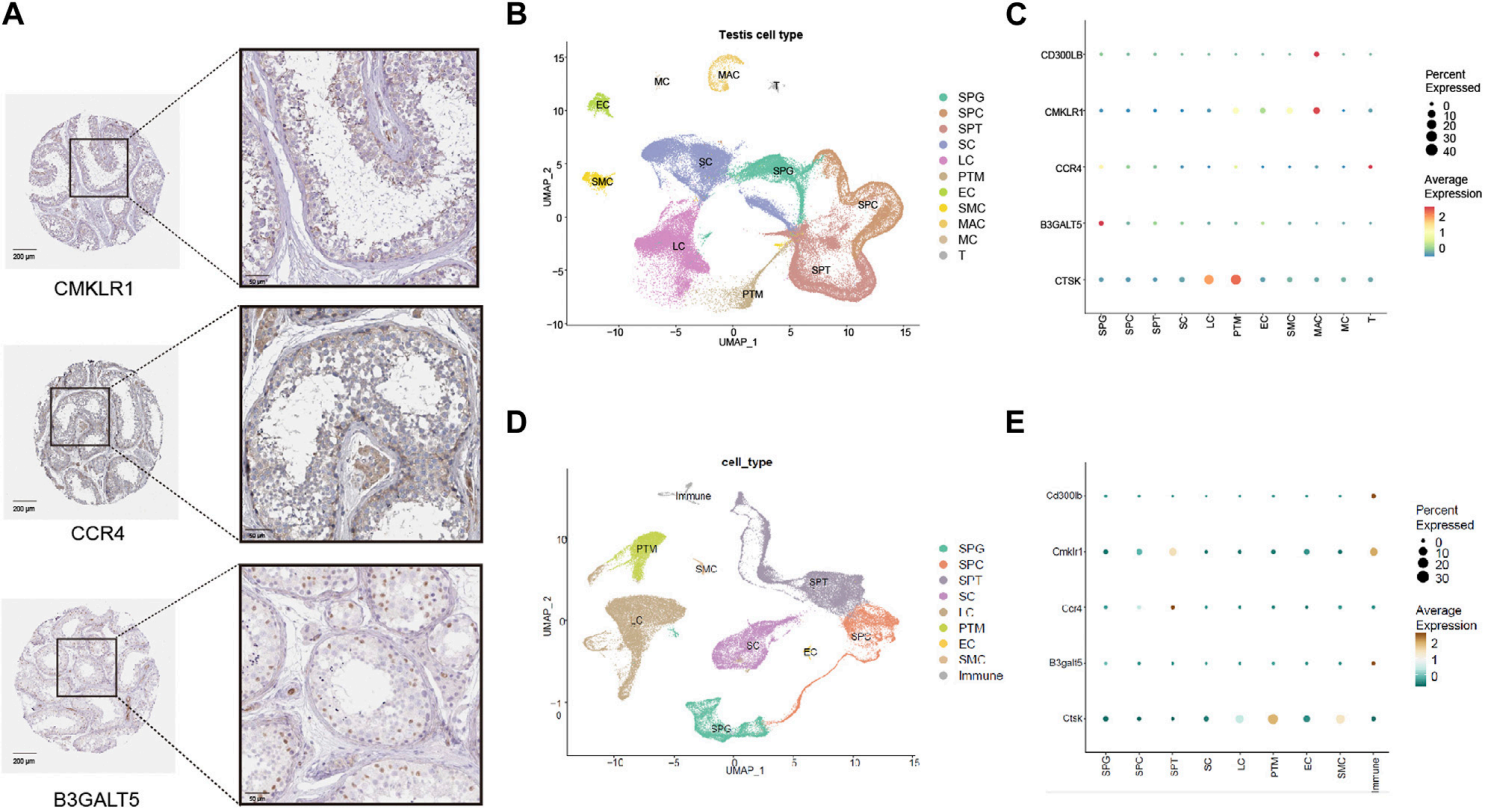
Expression patterns of five hub genes (*CD300LB, CMKLR1, CCR4, B3GALT5,* and *CTSK*) in the testicular tissues and cell types. **(A)** Immunohistochemistry results of five hub genes for healthy control in the HPA database (scale bars = 50 μm and 200 μm). **(B)** Cell type analysis of testicular cell for healthy male control. **(C)** Expression patterns of five hub genes categorized by human testicular cell types. **(D)** Cell type analysis of testicular cell for healthy male mice control. **(E)** Expression patterns of five hub genes categorized by mouse testicular cell types. HPA: Human Protein Atlas; SPG: spermatogonia; SPC: spermatocyte; SPT: spermatid/sperm; SC: Sertoli cell; LC: Leydig cell; PTM: peritubular myoid cell; EC: Endothelial cell; SMC: vascular smooth muscle cell; MAC: Macrophage; MC: Mast cell; T: T cell; Immune: immune cell.

To better reveal the condition of expression and localization for hub genes, we first explored the hub gene signature in the immunohistochemical data from the HPA database. As shown in Figure 5A, weak positive expression for CMKLR1 was mainly concentrated in the interstitial region and blood vessels beside spermatogenic tubules. For CCR4, weak positive staining was observed in the cytoplasmic region of germ cells near the basement membrane and Leydig cells. For B3GALT5, moderate positive expression was observed in the nuclei of germ cells near the basement membrane.

### 3.7 Examination of hub genes in single-cell transcriptome data

We investigated the expression pattern of cell types in healthy male controls with normal fertility. Various cell types in the testes from humans and mice were carefully classified according to a single-cell analysis algorithm (dimensional reduction by principal component analysis, cell clustering by FindClusters processing, and cluster annotation based on marker genes) (Figures 5B, D), and we found that hub genes had unique expression patterns in cell types. As shown in Figure 5C (human data), CMKLR1 and CD300LB were mainly enriched in macrophages due to their high average expression. CCR4 and B3GALT5 were both enriched in spermatogonia, while CTSK was predominantly enriched in Leydig cells and peritubular myoid cells. In Figure 5E (mouse data), Cd300lb and B3galt5 were strongly enriched in immune cells, and Cmklr1 and Ccr4 were preferentially enriched in spermatids/sperm. Ctsk was highly enriched in peritubular myoid cells. The data demonstrated that the hub genes may be closely related to the immune environment of the interstitial region and the entire process of spermatogenesis.

### 3.8 MiRNA-hub gene network

**FIGURE 6:**
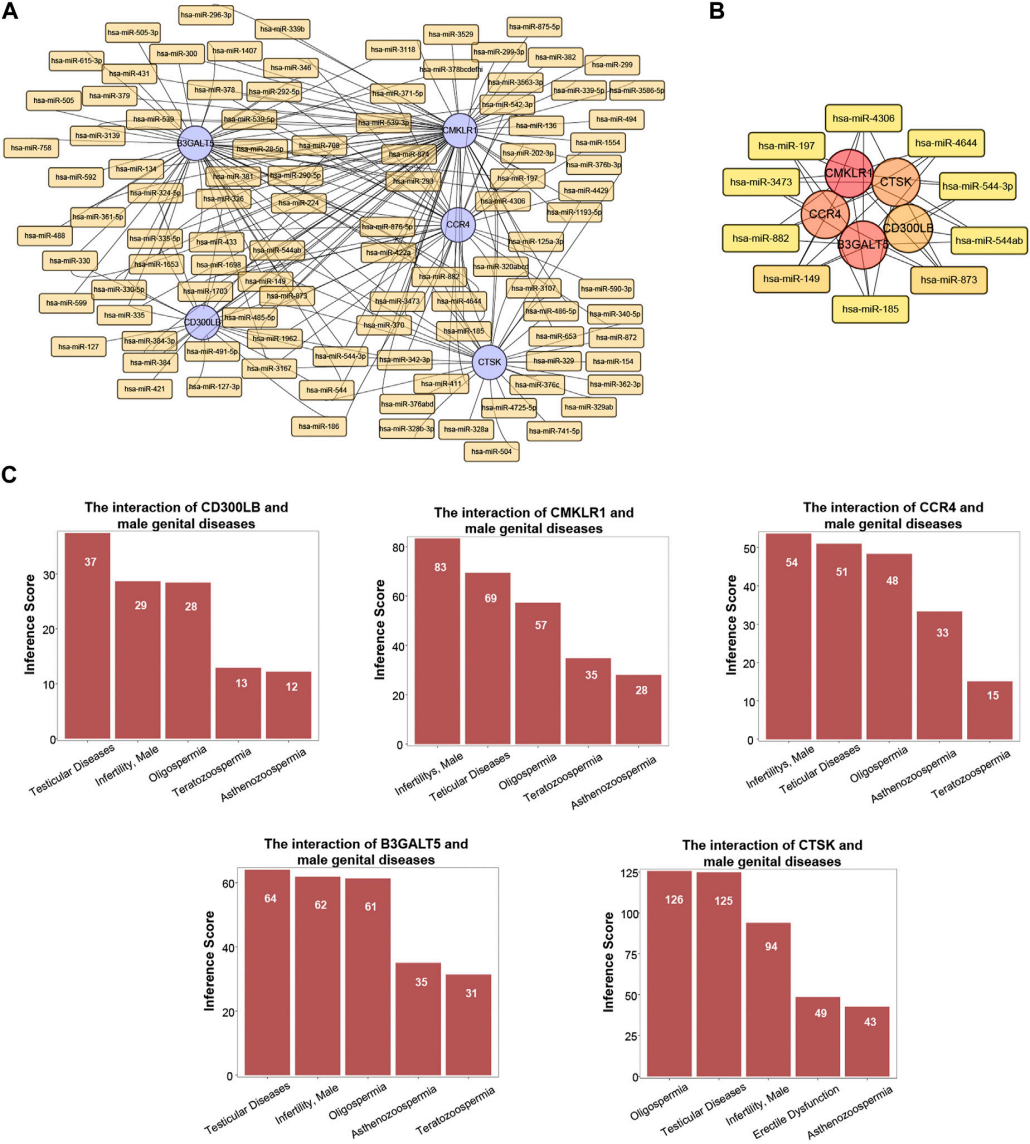
MiRNA profile and associated diseases related to five hub genes (*CD300LB, CMKLR1, CCR4, B3GALT5,* and *CTSK*). **(A)** MiRNA-hub gene regulatory network. **(B)** Top ten miRNA-hub gene regulatory network. **(C)** Association between five hub genes and infertility-related diseases.

MiRNAs have been revealed to play a critical role in male-related infertility disorders. Therefore, we selected the important miRNAs around the five hub genes in CE and AZS, and constructed a miRNA-hub gene regulatory network. A total of 224 miRNA-gene interactions were found from the miRcode database, and the miRNA profile is depicted in Figure 6A. Among these miRNAs, we selected the top ten important miRNAs that have more links to hub genes: hsa-miR-873, hsa-miR-149, hsa-miR-197, hsa-miR-4644, hsa-miR-4306, hsa-miR-3473, hsa-miR-882, hsa-miR-185, hsa-miR-544-3p, and hsa-miR-544ab. A total of 42 miRNA-hub gene interactions were identified in the network (Figure 6B). Among the top ten miRNAs, hsa-miR-149 and hsa-miR-873 were both linked to all five hub genes, while each of the other eight miRNAs was linked to four hub genes. These miRNAs may function as regulators in the development of AZS and CE.

### 3.9 Gene-disease association

Utilizing the CTD database, we demonstrated that the five hub genes (*CD300LB, CMKLR1, CCR4, B3GALT5,* and *CTSK*) significantly affected male reproductive system diseases. The male reproductive system diseases were focused on testicular diseases, infertility (male), asthenozoospermia, oligospermia, and teratozoospermia (Figure 6C).

### 3.10 Immune infiltration correlation

**FIGURE 7:**
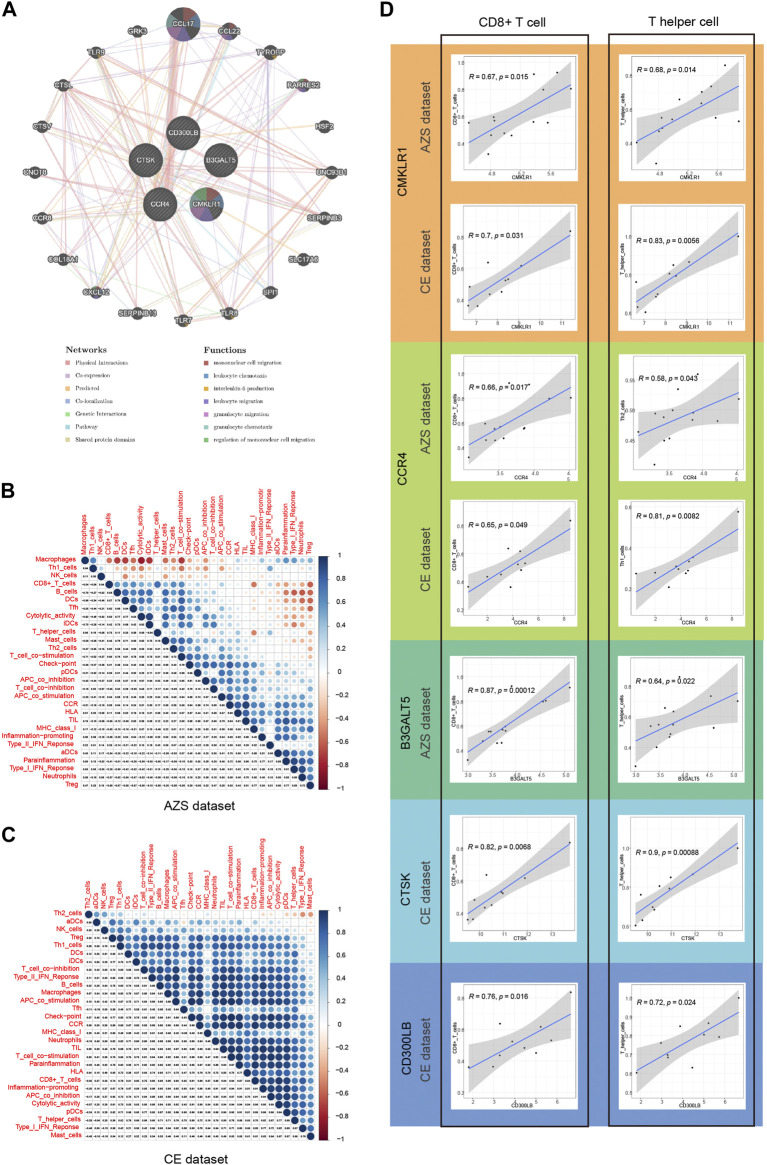
Correlation of five hub genes (*CD300LB, CMKLR1, CCR4, B3GALT5,* and *CTSK*) with immune infiltration and gene-gene interaction. **(A)** Gene–gene interaction network for five hub genes based on the GeneMANIA database. **(B)** Correlation results of immune cell/function in AZS samples, and the spearman correlation coefficient is represented by the dot diameter and color bar. **(C)** Correlation results of immune cell/function in CE samples. **(D)** Correlation results between five hub genes and two main immune cell types (CD8^+^ T cell and T helper cell). AZS: asthenozoospermia; CE: chronic epididymitis; ssGSEA: single-sample gene set enrichment analysis.

Before analyzing immune infiltration, we analyzed the correlation between hub genes and their interacting genes. The gene‒gene interaction results indicated that their gene functions were enriched in mononuclear cell migration, leukocyte chemotaxis, interleukin-6 production, leukocyte migration, granulocyte migration, and so on (Figure 7A).

Single-sample GSEA is a very well-established computational algorithm for calculating immune infiltration for genetic matrices. After calculating the score of 29 immune cells/functions for each sample, we identified the correlation between the 29 immune cells/functions in AZS, which is in Figure 7B, while the correlation in CE is shown in Figure 7C. In the CE dataset, most immune cells/functions had a positive influence on each other. The situation (positive influence) was the same for AZS, except for macrophages, Th1 cells, and NK cells.

Next, we further explored the correlation between the hub genes and immune infiltration. As shown in Figure 7D, for AZS samples, hub genes were significantly linked to the level of CD8^+^ cell infiltration (CMKLR1, *r* = 0.67, *p* = 0.015; CCR4, *r* = 0.66, *p* = 0.017; B3GALT5, *r* = 0.87, *p* = 0.00012) and T helper cell infiltration (CMKLR1, *r* = 0.68, *p* = 0.014; CCR4, *r* = 0.58, *p* = 0.043; B3GALT5: *r* = 0.64, *p* = 0.022). For CE samples, hub genes had an evident correlation with CD8^+^ cell infiltration (CMKLR1, *r* = 0.7, *p* = 0.031; CCR4, *r* = 0.65, *p* = 0.049; CTSK: *r* = 0.82, *p* = 0.0068; CD300LB, *r* = 0.76, *p* = 0.016) and T helper cells (CMKLR1, *r* = 0.83, *p* = 0.0056; CCR4, *r* = 0.81, *p* = 0.0082; CTSK: *r* = 0.9, *p* = 0.00088; CD300LB, *r* = 0.72, *p* = 0.024). These results suggested that CD8^+^ cells and T helper cells may act as important immune cells regulating the progression of inflammation in CE and impaired sperm function.

### 3.11 Experimental examination of sperm specimens

**FIGURE 8:**
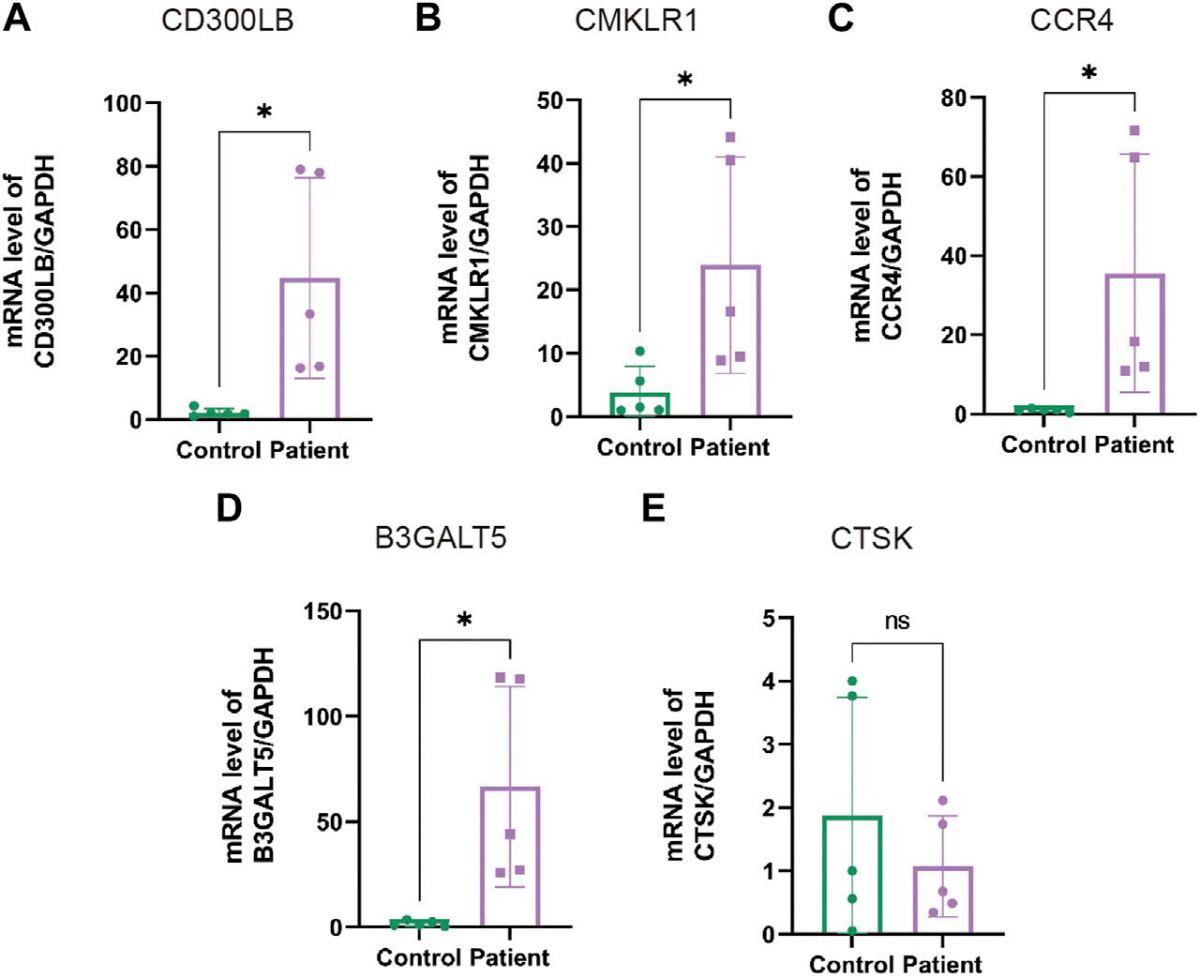
The mRNA levels of hub genes in sperm specimens measured by qRT-PCR. The mRNA levels of CD300LB **(A)**, CMKLR1 **(B)**, CCR4 **(C)**, B3GALT5 **(D)**, and CTSK **(E)** in the patients with AZS and CE, as well as healthy controls, with GAPDH serving as the standard control. * indicates *p* < 0.05. qRT-PCR: quantitative real-time polymerase chain reaction; AZS: asthenozoospermia; CE: chronic epididymitis; ns: not significant.

In the next step, we performed qRT-PCR to examine the expression of hub genes between the patients with AZS and CE and healthy controls. As shown in Supplementary Table S4, patients with AZS and CE had decreased levels of sperm progressive motility and increased levels of seminal elastase compared with the control group. Quantitative RT-PCR results indicated that there were evident differences in hub genes between the patient and control groups (*p* < 0.05), and the mRNA levels of hub genes (*CD300LB, CMKLR1, CCR4,* and *B3GALT5*) were significantly elevated in the sperm affected by AZS and CE (Figure 8). However, the mRNA level of CTSK was not statistically significant. The data synergistically validated that these hub genes shared the same expression trend in the sequencing results and experimental results.

## 4 Discussion

Research has indicated that male infertility has been continuously rising over the past few decades (Sansone et al., 2018). Inflammation in the male genital tract is known to exert a negative effect on normal sperm viability and motility. This is a good explanation for the high incidence (up to 40%) of male infertility in epididymitis. Chronic epididymitis, a common chronic urogenital inflammatory disease, is often secondary to urological infections such as orchitis, prostatitis, vesiculitis, bladder cystitis and other infectious diseases of the urogenital tract. In humans, the epididymis is a duct-like structure that links the testis to the vas deferens. It has four anatomical regions: the initial segment, caput, corpus, and cauda. During epididymal transit, the immature spermatozoa produced by the testis acquire maturation, motility and fertilizing capabilities (James et al., 2020). Additionally, chronic inflammation inevitably affects the normal secretory function of the epididymis (Condorelli et al., 2017). Chemokine release, inflammatory mediator action, and immune response activation are jointly involved in the development of impaired sperm functions (Silva et al., 2018).

Although evidence has revealed that inflammation/immunity closely correlates with CE-associated AZS, the specific mechanisms and molecular alterations are still not clear. Bioinformatics has a distinct advantage in processing large volumes of transcriptome data/RNA-seq data and analyzing potentially valuable pathways. For example, the genetic profile from transcriptome data was systematically studied in the subtype of Sertoli cell-only syndrome (SCOS), and several genes were implicated in the pathogenesis of Sertoli cell-only syndrome (Kusz-Zamelczyk et al., 2013; Chen et al., 2022). Based on the above theories, we used multiomics analysis to assess the genetic landscape and immune infiltration in AZS and CE. First, after the combined screening of the 2256 AZS DEGs and 1742 CE DEGs, a total of 109 intersecting DEGs were found. We observed that the intersecting genes only accounted for a small part of DEGs in a single disease. However, these genes may have important functions.

In the functional enrichment results, significant biological processes and pathways, such as inflammatory response (GO), immune response (GO), neutrophil degranulation (REACTOME), and interleukin-4 and interleukin-13 signaling (REACTOME), were identified. Regarding the immune response, the epididymal epithelium was reported to have the ability to release and produce cytokines that attract inflammatory cells through TLR2 activation by sensing the presence of pathogens (Schleimer et al., 2007; Zhao et al., 2008). Moreover, in response to uropathogenic *Escherichia coli* infection, TLR4 and TLR5 together trigger epididymal innate immune responses (Anders and Patole, 2005; Andersen-Nissen et al., 2007; Cheng et al., 2016). Excess neutrophil degranulation is very common in many inflammatory disorders, such as severe asphyxic episodes of asthma (Lacy, 2006). Ochsendorf et al. (Ochsendorf, 1999) revealed that neutrophils are the main source of ROS, and the oxidative burst from neutrophils functions as a disruptor of sperm capacitation and the acrosome reaction. For interleukin-4 and interleukin-13 signaling, the anti-inflammatory and wound-healing properties of macrophages are alternately stimulated with either IL-4 or IL-13. Maresz et al. (Maresz et al., 2008) found that by preserving a population of macrophages that are variably activated, IL-13 contributed to testicular immune exclusivity. In summary, immune-related biological processes and inflammatory cell/factor aggregation synergistically participate in the pathological mechanism of CE-associated AZS.

According to the diagnostic evaluation of five hub genes in the AZS, CE, and validation sets, *CMKLR1, CCR4, B3GALT5, CD300LB,* and *CTSK* were selected as hub genes for both AZS and CE (AUC >0.7). Furthermore, immune infiltration analysis indicated that these hub genes were positively related to the levels of CD8^+^ T cells and/or T helper cells (CD4^+^ T cells). CMKLR1 (chemerin chemokine-like receptor 1) was found to be preferentially expressed in T cell subsets (Jeong et al., 2022). Estienne et al. (Estienne et al., 2020) demonstrated that chemerin (the ligand of CMKLR1) in seminal plasma had a negative relationship with rooster spermatozoa motility. Xu et al. (Xu, 2022, 1) reported that CD8^+^ T cells were closely linked to CMKLR1 and WTAP in the immune cell infiltration of pancreatic ductal adenocarcinoma. An *in vivo* study confirmed that knockout of CMKLR1 in mice resulted in inhibition of activation of CD4^+^ T cells, as well as attenuation of recruitment of inflammatory cells (e.g., CD8^+^ T cells) induced by pulmonary inflammation (Demoor et al., 2011). Thus, CMKLR1 may serve as an important immunomodulator driving inflammation and tumor immunity, and its immunological role was first reported in male infertility. CD300LB (CD300 molecule like family member b) is a non-canonical immunoglobulin (Ig) superfamily activating receptor (Martínez-Barriocanal et al., 2010). Studies have indicated that CD300LB is expressed in dendritic cells (DCs) and controls the biological function of DCs (Clark et al., 2009; Gasiorowski et al., 2013). Additionally, high CD300LB expression in CD4^+^ T cells mediates the inflammatory response in patients with systemic lupus erythematosus and heart failure (Zhao et al., 2014; Abplanalp et al., 2021). However, the function of CD300LB in male germ cells is not clear, and it is worth studying further.

Regarding CCR4 (C-C motif chemokine receptor 4), researchers found that depletion of the CCR4-associated protein CAF1 could contribute to abnormal spermatogenesis in male mice (Berthet et al., 2004), and peripheral CCR4+ FOXP3+ T cells were remarkably lower in women with fertility problems than in healthy controls (Diao et al., 2017). Targeting CCR4 was shown to be able to improve immunity by preventing Treg (CD4^+^) entry into the tumor microenvironment, and subsequently overcome sorafenib resistance (Curiel et al., 2004; Gao et al., 2022). In an *in vivo* study, CTSK (cathepsin K, a lysosomal cysteine proteinase) was found to be a significantly upregulated gene involved in the pathogenesis of male-derived sterility in cattle yaks (Cao et al., 2022). The genetic alteration of CTSK is responsible for interleukin-18 mediated CD4^+^ T-cell differentiation in arthritis-associated disease (Min et al., 2021). B3GALT5 (beta-1,3-galactosyltransferase 5), a member of a family of membrane-bound glycoproteins, was enhanced in pancreatic malignancies (Aronica et al., 2017). Ana et al. (Magalhães et al., 2015) suggested that upregulation of B3GALT5 positively correlates with local proinflammatory features in the gastric niche. Based on these findings, all five hub genes were proven to exert functions in spermatogenesis and/or the immune response to some extent, but the reports regarding the role of the hub genes in the immune environment of reproductive system inflammation are very limited. Our study identified the association between hub genes and immune infiltration (especially for CD8^+^ T cells and T helper cells), and indicated that these factors may have potential as biomarkers of CE-associated AZS.

MiRNA play a vital role in male infertility (Barbu et al., 2021). The seminal fluid was discovered to contain many microRNAs, and the change in miRNA profile had a close relationship with AZS and epididymitis (Barbu et al., 2021; Gong et al., 2022). In our study, hsa-miR-149, 197, and 185 were selected as some of the top ten key miRNAs based on miRNA-hub gene interactions. A recent study found that sperm-borne miR-149 could be used as a predictor of good-quality embryos. The lower the expression level of hsa-miR-149, the better the quality of the embryo (Li et al., 2021). MiR-197 is expressed in the majority of spermatogenic cells, and it is enriched in the phenotype of SCOS and meiotic arrest (Bai et al., 2022). In addition, researchers used a luciferase reporter gene experiment to prove that miR-185 targets CTSK mRNA (Li et al., 2019). Our data built on the above findings and further confirmed the involvement of these miRNAs in the molecular landscape of asthenozoospermia and chronic epididymitis.

Although the genetic signature was characterized through genomic datasets, an independent validation set, immunohistochemical staining, and experimental results, there are still several limitations in this study. Our data are subject to some bias in the analysis due to different sequencing techniques and different tissue sources. Uniform sequencing technology and tissue sources will facilitate bias reduction. Second, because the subjects had scrotal pain and inflammation in the epididymis, epididymal tissue acquisition was very difficult at this time, and may further aggravate inflammation. Third, although we examined the expression condition of hub genes by qRT-PCR, histological expression analysis and in-depth mechanistic exploration were not well performed. Elucidating the specific expression and potential mechanism is very important and needs to be carried out in our future investigation.

## 5 Conclusion

In summary, we investigated the genetic landscape of AZS and CE and elucidated the hub gene signature (*CD300LB, CMKLR1, CCR4, B3GALT5,* and *CTSK*) and specific biological pathways. The immune response mediated by CD8^+^ and T helper cells affected the progression of AZS and CE. Our results provide novel insight into potential biomarkers and therapeutic targets of CE-associated AZS.

## Data Availability

The original contributions presented in the study are included in the article/Supplementary Materials, further inquiries can be directed to the corresponding authors.
