# Supplementary material for: Identification of hub genes and biological mechanisms underlying the pathogenesis of asthenozoospermia and chronic epididymitis
Source: Front Genet. 2023 Apr 21;14:1110218. doi: 10.3389/fgene.2023.1110218 (PMC10160426; doi:10.3389/fgene.2023.1110218)
Supplement: Supplementary file 1 [file Table1.DOCX]

**Supplementary table 1**

GEO datasets analyzed in the study.

| Dataset | | Numbers of participants  (Patient/Control) | Platform | Last update date | Country/Region |
| --- | --- | --- | --- | --- | --- |
| Asthenozoospermia | |  |  |  |  |
|  | GSE160749 | 11 (5/6) | GPL17692 | Nov 05, 2021 | Czech Republic |
|  | GSE22331 | 60 (30/30) (mixed samples) | GPL570 | Jun 14, 2010 | China |
|  | GSE26881 | 12 (3/9) | GPL6244 | Jul 26, 2018 | USA |
| Chronic epididymitis | |  |  |  |  |
|  | GSE199903 | 10(5/5) | GPL16791 | Aug 01, 2022 | Hong Kong, China |
| Single-cell transcriptome (healthy control) | |  |  |  |  |
|  | GSE149512 | 10 (Control) | GPL24676/ GPL27644 | Feb 03, 2021 | China |

GEO: Gene Expression Omnibus; GSE: GEO Series; GPL: GEO Platform.

|  |
| --- |
