# Supplementary material for: Identification of hub genes and biological mechanisms underlying the pathogenesis of asthenozoospermia and chronic epididymitis
Source: Front Genet. 2023 Apr 21;14:1110218. doi: 10.3389/fgene.2023.1110218 (PMC10160426; doi:10.3389/fgene.2023.1110218)
Supplement: Supplementary file 2 [file Table2.DOCX]

**Supplementary table 2**

Resource addresses of immunohistochemical data for three hub genes in the HPA database.

| Gene | URL | Protein Atlas version |
| --- | --- | --- |
| CMKLR1 | https://www.proteinatlas.org/ENSG00000174600-CMKLR1/tissue/testis#img | Version 21.0 |
| CCR4 | https://www.proteinatlas.org/ENSG00000183813-CCR4/tissue/testis#img | Version 21.0 |
| B3GALT5 | https://www.proteinatlas.org/ENSG00000183778-B3GALT5/tissue/testis#img | Version 21.0 |

HPA database: Human Protein Atlas database; URL: uniform resource locator.
