# Supplementary material for: Identification of hub genes and biological mechanisms underlying the pathogenesis of asthenozoospermia and chronic epididymitis
Source: Front Genet. 2023 Apr 21;14:1110218. doi: 10.3389/fgene.2023.1110218 (PMC10160426; doi:10.3389/fgene.2023.1110218)
Supplement: Supplementary file 3 [file Table3.DOCX]

**Supplementary table 3**

Primer sequences used in qRT-PCR analysis.

| Gene | Primer type | Sequence/Target sequence |
| --- | --- | --- |
| CD300LB (human) | Forward | 5’- GGTCCCTGACGGTTCAATG -3’ |
| CD300LB (human) | Reverse | 5’- GATGGACACACGGTCACTCT -3’ |
| CMKLR1 (human) | Forward | 5’- TGGACTACCACTGGGTTTTCG -3’ |
| CMKLR1 (human) | Reverse | 5’- CGAGAGATGGGGAACTCAAGAAG -3’ |
| CCR4 (human) | Forward | 5’-AGAAGGCATCAAGGCATTTGG-3’ |
| CCR4 (human) | Reverse | 5’-ACACATCAGTCATGGACCTGAG-3’ |
| B3GALT5 (human) | Forward | 5’-AAGACCATGATGGGCATAGAATG-3’ |
| B3GALT5 (human) | Reverse | 5’-CCTGTCCCACGGATATTCAGAT-3’ |
| CTSK (human) | Forward | 5’-ACACCCACTGGGAGCTATG-3’ |
| CTSK (human) | Reverse | 5’-GACAGGGGTACTTTGAGTCCA-3’ |
| GAPDH (human) | Forward | 5’- TGGTCACCAGGGCTGCTTTTA -3’ |
| GAPDH (human) | Reverse | 5’- CATCGCCCCACTTGATTTTG -3’ |

qRT-PCR: quantitative real-time polymerase chain reaction; CD300LB: CD300 molecule like family member b; CMKLR1: chemerin chemokine-like receptor 1; CCR4: C-C motif chemokine receptor 4; B3GALT5: beta-1,3-galactosyltransferase 5; CTSK: cathepsin K; GAPDH: glyceraldehyde-3-phosphate dehydrogenase.
