# Supplementary material for: Identification of hub genes and biological mechanisms underlying the pathogenesis of asthenozoospermia and chronic epididymitis
Source: Front Genet. 2023 Apr 21;14:1110218. doi: 10.3389/fgene.2023.1110218 (PMC10160426; doi:10.3389/fgene.2023.1110218)
Supplement: Supplementary file 4 [file Table4.DOCX]

**Supplementary table 4**

Demographic characteristics and laboratory examinations of participants in the study.

|  | AZS and CE | Control |
| --- | --- | --- |
| Age (year) | 29.4±5.3 | 30.4±5.0 |
| BMI (kg m^-2^) | 22.8±1.7 | 24.0±2.5 |
| Testicular volume (ml) | 13.3±1.0 | 13.4±1.0 |
| Karyotype | 46, XY | 46, XY |
| Serum FSH (mIU ml^-1^) | 5.8±1.8 | 5.5±1.0 |
| Serum LH (mIU ml^-1^) | 4.9±1.2 | 4.2±1.0 |
| Serum T (ng ml^-1^) | 3.8±1.0 | 3.8±1.3 |
| Serum E_2_ (pg ml^-1^) | 26.3±3.9 | 27.6±5.7 |
| Sperm concentration (10^6^ ml^-1^) | 12.7±3.7 | 26.3±7.9 |
| Sperm progressive motility (%) | 16.4±7.4 | 46.9±8.9 |
| Nomal sperm morphology (%) | 2.9±0.8 | 5.5±0.9 |
| Seminal elastase (ng/ml) | 2596.0±804.1 | 335.2±170.9 |
| History of scrotal pain | Pos | Neg |
| Duration of CE (months) | 6.2±3.5 | - |
| Ultrasound findings | Enlarged epididymis (n=4), Enlarged epididymis with uneven internal echogenicity (n=1) | - |
| Treatment of CE | Antibiotic (n=5) | - |
| History of varicocele | Neg | Neg |
| History of diabetes mellitus | Neg | Neg |
| History of scrotal trauma | Neg | Neg |
| Exposure to radiation and environmental pollutants | Neg | Neg |
| Smoking | Neg | Neg |
| Alcohol abuse | Neg | Neg |

Testicular volume represents the mean value of bilateral testicular volume.

AZS: Asthenozoospermia; CE: Chronic epididymitis; BMI: body mass index; FSH: follicle‑stimulating hormone (normal range: 1.27-19.26 mIU ml^-1^); LH: luteinizing hormone (normal range: 1.24-8.62 mIU ml^-1^); T: testosterone (normal range: 1.75-7.81 ng ml^-1^); E_2_: estradiol (normal range: ≤ 38.95 pg ml^-1^); Pos: positive; Neg: negative.
